# Benefits and Challenges of Applying Bacteriophage Biocontrol in the Consumer Water Cycle

**DOI:** 10.3390/microorganisms12061163

**Published:** 2024-06-07

**Authors:** Brandon Reyneke, Benjamin Havenga, Monique Waso-Reyneke, Sehaam Khan, Wesaal Khan

**Affiliations:** 1 Department of Microbiology, Faculty of Science, Stellenbosch University, Private Bag X1, Stellenbosch 7602, South Africa; 2 Faculty of Health Sciences, University of Johannesburg, P.O. Box 17011, Doornfontein 2028, South Africa

**Keywords:** bacteriophages, biocontrol, consumer water cycle, bacterial pathogens

## Abstract

Bacteria (including disinfection- and antibiotic-resistant bacteria) are abundant in the consumer water cycle, where they may cause disease, and lead to biofouling and infrastructure damage in distributions systems, subsequently resulting in significant economic losses. Bacteriophages and their associated enzymes may then offer a biological control solution for application within the water sector. Lytic bacteriophages are of particular interest as biocontrol agents as their narrow host range can be exploited for the targeted removal of specific bacteria in a designated environment. Bacteriophages can also be used to improve processes such as wastewater treatment, while bacteriophage-derived enzymes can be applied to combat biofouling based on their effectiveness against preformed biofilms. However, the host range, environmental stability, bacteriophage resistance and biosafety risks are some of the factors that need to be considered prior to the large-scale application of these bacterial viruses. Characteristics of bacteriophages that highlight their potential as biocontrol agents are thus outlined in this review, as well as the potential application of bacteriophage biocontrol throughout the consumer water cycle. Additionally, the limitations of bacteriophage biocontrol and corresponding mitigation strategies are outlined, including the use of engineered bacteriophages for improved host ranges, environmental stability and the antimicrobial re-sensitisation of bacteria. Finally, the potential public and environmental risks associated with large-scale bacteriophage biocontrol application are considered, and alternative applications of bacteriophages to enhance the functioning of the consumer water cycle, including their use as water quality or treatment indicators and microbial source tracking markers, are discussed.

## 1. Introduction

Globally, the agricultural, municipal, mining, industrial, afforestation and energy sectors are recognised as the primary potable and freshwater consumers and form part of the larger consumer water cycle (i.e., environmental water extraction, treatment, storage, distribution, wastewater treatment and removal) [1]. Governmental organisations are thus actively promoting the use of alternative water sources and the optimisation of the consumer water cycle (e.g., direct potable reuse of treated wastewater) in order to directly increase global water security [2,3] and effectively achieve Sustainable Development Goal 6—clean water and sanitation for all [4]. However, while increased concentrations of traditional chemical contaminants (e.g., heavy metals) and contaminants of emerging concern (e.g., micropollutants, pharmaceuticals and personal care products) may impact public health, the microbial contaminants prevalent throughout the consumer water cycle do not only pose a health risk to the end-users, but also contribute to the decline of water-based infrastructure (i.e., biofouling and corrosion) and decrease system or process efficiency in various industries (e.g., food or energy sector) [5,6]. Treatment strategies such as membrane ultrafiltration, reverse osmosis, ultraviolet treatment and chlorination have thus been implemented throughout the industrial and municipal sectors to mitigate traditional chemical and microbial contaminants within urban and rural water systems.

Many of the conventional disinfection or microbial control approaches employed are, however, inadequate in combatting the rising complexity associated with the persistence of microbial contaminants, primarily due to the ability of these microorganisms to gain resistance to the intervention strategies [7]. For example, resistance to conventional disinfectants containing chlorine has been observed amongst bacteria such as *Mycobacterium*, *Bacillus*, *Legionella*, *Pseudomonas* and *Sphingomonas*, which are generally present in water treatment and reclamation systems [8]. Additionally, commonly used treatment strategies are not always effective in penetrating and eliminating microbial biofilms (an assemblage of surface-associated microbial cells enclosed in an extracellular polymeric substance matrix), which commonly form at the liquid–surface interface, and may contain microbial pathogens and contribute to the persistence of microbial contaminants in different components of the water treatment, distribution and reclamation systems [9].

Thus, as (1) the use of chemical disinfection strategies has been curtailed by regulations aimed at minimising the formation of harmful disinfection by-products, (2) the use of multiple-barrier physical treatment strategies may be energy-consuming and costly, and (3) the implementation of non-targeted treatment interventions may negatively influence microbial dynamics in biological processes, there is a need for sustainable and environmentally friendly treatment strategies to be implemented for the targeted removal of microbial contaminants in complex environments.

The biocontrol potential of lytic bacteriophages (viruses that infect and lyse a target bacterial host) has been widely recognised, and they have been employed in the food industry to target food-borne pathogens, for the removal of biofilms in the food and medical industry and for the treatment of infectious diseases in both human and veterinary medicine [9,10,11]. Bacteriophages could also be applied in biological control strategies to reduce economic losses in agriculture by targeting plant pathogens and aquaculture by targeting fish pathogens, or in bioremediation strategies for the selective removal of bacteria from water [11,12,13,14].

In this review, the bacteriophage life cycle and associated characteristics that highlight their potential as biocontrol agents is outlined. Additionally, the opportunities for the application of bacteriophage biocontrol in various industries throughout the consumer water cycle are discussed, whereafter certain limitations of bacteriophage biocontrol and corresponding mitigation strategies are outlined. Additionally, the potential public and environmental risks associated with the large-scale bacteriophage biocontrol application are considered and biosafety recommendations are presented. Lastly, we focus on the alternative application of bacteriophages to enhance the functioning of the consumer water cycle, including their use as water quality or treatment indicators and microbial source tracking (MST) markers.

## 2. Bacteriophage Characteristics Highlighting Their Potential as Biocontrol Agents

### 2.1. Life Cycle and Host Specificity

**Figure 1:**
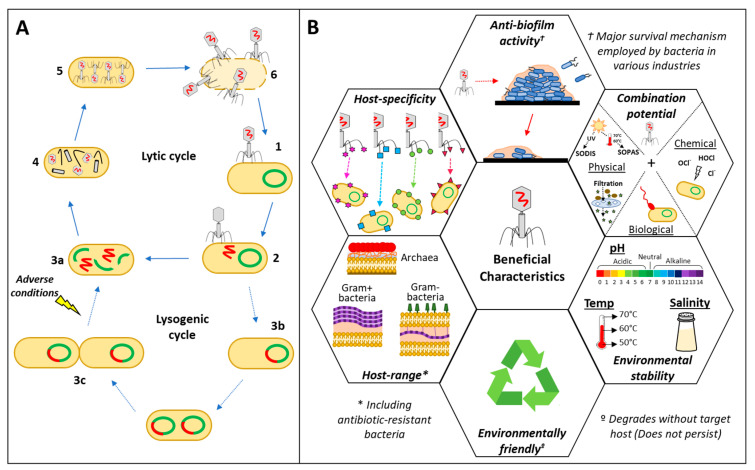
(**A**) Different bacteriophage life cycles and (**B**) beneficial bacteriophage characteristics highlighting their potential as biocontrol agents in the consumer water cycle.

**Figure 2:**
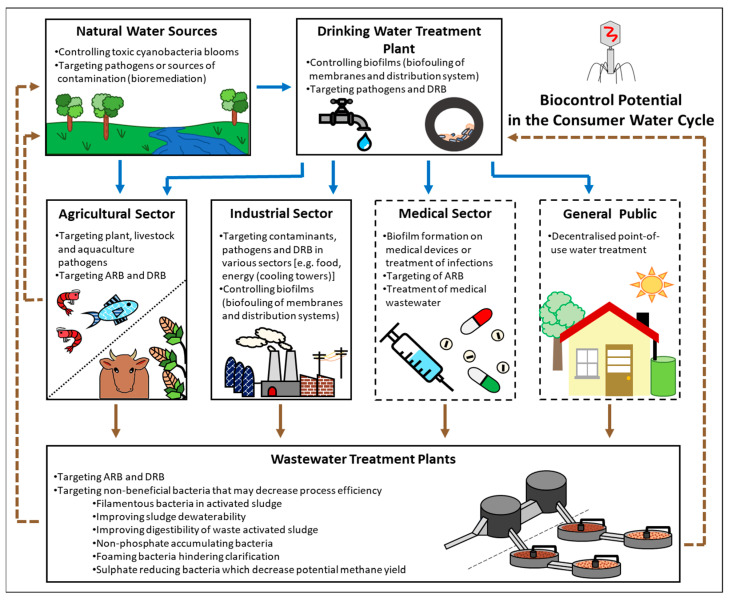
Sectors in the consumer water cycle where bacteriophage biocontrol can be applied for the targeted removal of problematic bacteria/biofilms.

**Figure 3:**
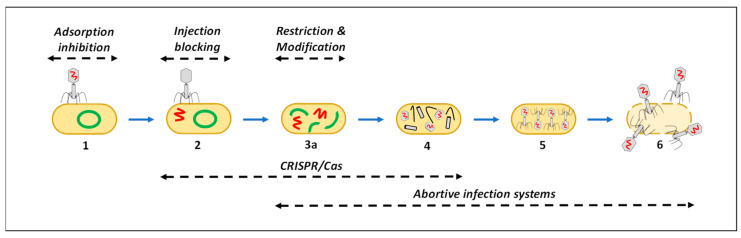
Dominant bacteriophage resistance mechanisms employed by bacteria (adapted from Frampton et al. [13], Labrie et al. [16] and Samson et al. [54]).

Bacteriophages (also referred to as “phages”) are bacterial viruses that are ubiquitously distributed in the environment (especially in aquatic ecosystems) and are considered the most abundant “life forms” on earth (population of approximately 10^31^) [15]. They play an important role in ecology by infecting and killing bacteria in one of two ways [16]. Bacteriophages may exert their antibacterial activity by either (1) infecting, replicating and lysing the host bacterial cell (lytic/virulent phages) [Figure 1A; (1, 2, 3a, 4 and 6)] or (2) integrating their genome into the host genome (now referred to as a prophage) and, in response to external stressors/environmental cues, initiating bacteriophage replication and causing bacterial cell lysis (lysogenic/temperate phages) [15] [Figure 1A; (1, 2, 3b, 3c and 3a)]. While lysogeny has subsequently been identified as a survival strategy for not only the bacteriophage (prophage) but also its lysogenic host, and lysogenic hosts frequently outcompete their non-lysogenic host counterparts due to the competitive advantage the prophage genes confer (e.g., antibiotic resistance), lytic bacteriophages display ideal characteristics for use as bacterial biocontrol agents [13].

The basic bacteriophage virion consists of a protein envelope containing a specific nucleic acid [single- or double-stranded ribonucleic acid (RNA) or deoxyribonucleic acid (DNA)], while certain bacteriophages have also been shown to contain lipids in the envelope or as part of a particular lipid wall [17]. The high host-specificity of bacteriophages is attributed to proteins at the tip of the tail fibres selectively binding to receptors on the bacterial cell surface (e.g., the capsule, lipopolysaccharides, transport proteins, flagella and pili) (Figure 1B) during the initial adsorption step [Figure 1A (1)] [18]. While most bacteriophages are species- or strain-specific, due to their affinity for certain binding receptors, some bacteriophages (termed polyvalent bacteriophages) have been shown to exhibit a relatively broad host range due to relaxed receptor binding specificity or their ability to recognise and attach to multiple receptors on different bacterial species or genera (Figure 1B) [19]. Thus, while bacteriophages exhibiting high host-specificity could potentially be applied for the targeted removal of a specific pathogen, polyvalent bacteriophages may be used in instances where multiple bacterial contaminants need to be removed or eliminated.

### 2.2. Bacteriophage Enzymes and Potential for Combination Applications

At the completion of the bacteriophage life cycle, host bacterial cell lysis and the subsequent release of bacteriophage progeny [Figure 1A (6)] is facilitated by a combination of bacteriophage enzymes, including holins, endolysins and spanins [13,20].

Specifically, holins form pores in the bacterial cell membrane, facilitating the entry of endolysins into the periplasm of the bacterial cell where they degrade the bacterial cell wall by hydrolysing the glycosidic or peptide bonds in peptidoglycan. Spanins are then able to fuse the inner and outer bacterial cell membrane to form pores from which the bacteriophage progeny are released [13,20]. Due to the non-selective membrane disruption (i.e., enzymes cause non-selective pore formation) caused by the bacteriophage enzymes, bacteriophages have been shown to be efficient in eradicating both antibiotic-resistant bacteria (ARB) and disinfection-resistant bacteria (DRB) [21,22]. Additionally, bacteriophage enzymes (e.g., polysaccharide depolymerising enzymes) facilitate the degradation of the extracellular matrix that encapsulates bacterial biofilms, allowing bacteriophages to infect and eradicate these complex microbial community structures (Figure 1B) [9]. For example, Meng et al. [23] combined a bacteriophage lysin (LySMP) with a bacteriophage and antibiotics to disrupt *Staphylococcus suis* biofilms. The results show that the LySMP could disrupt the biofilms by >80%, while the bacteriophage and antibiotic-only treatments were ineffective in disrupting the pre-formed biofilms. Additionally, when LySMP was combined with ampicillin, amoxicillin and ciprofloxacin, synergistic activity was observed with a significant eradication of viable cells.

In contrast, Zhang and Hu [24] combined bacteriophages (mixture of RNA bacteriophages) with a chlorination treatment to inhibit *Pseudomonas aeruginosa* biofilm formation. The bacteriophage-only treatment (10^3^ to 10^7^ phages/mL) inhibited *P. aeruginosa* biofilm formation by 30 to 81% and reduced pre-existing biofilms by 36 to 80%. In comparison, while the chlorination treatment (210 mg/L) reduced biofilm formation by 83 to 89%, it showed no activity against the pre-existing biofilms. However, in comparison to the individual treatments, a combination of the RNA bacteriophages (10^7^ phages/mL) and chlorine (210 mg/L) reduced both biofilm formation (94 ± 2%) and pre-existing biofilms (88 ± 6%), thereby significantly increasing the observed anti-biofilm and anti-adhesive activity.

The synergistic or increased treatment efficiency that may be obtained when combining bacteriophage biocontrol with conventional treatment strategies is thus a crucial characteristic that highlights the potential of bacteriophages to eliminate microbial contaminants throughout the consumer water cycle (Figure 1B). Subsequently, the combination of bacteriophages with conventional antibiotics [25,26], chemical disinfectants [24], filtration systems [27,28], solar-based treatment [29,30,31], and the use of bacteriophages to enhance various industrial or water treatment processes have been investigated [12,32] (Outlined in Section 3). Additionally, while most biocontrol strategies are concentration-dependent and the treatment concentration may decrease after dosage, the bacteriophage treatment concentration will increase with time, as the bacteriophages continue to replicate and infect the target bacteria [15]. Moreover, once the target bacteria have been eliminated and the bacteriophage no longer has a specific host to infect, the bacteriophage will eventually disintegrate. Bacteriophage biocontrol thus has the potential to serve as an environmentally friendly treatment intervention, as the bacteriophages will not survive and proliferate without the required target host (Figure 1B) [15].

### 2.3. Environmental Stability

In addition to their host specificity and the enzymes they produce, the environmental stability that bacteriophages display also highlights their potential as biocontrol agents in various aquatic environments or in biotechnological processes throughout the consumer water cycle (Figure 1B). However, research has shown that the influences of these external chemical and physical factors (e.g., pH, temperature, UV, ions and salinity) are highly diversified amongst not only bacteriophage families, but also within the specific families [17]. This may primarily be attributed to factors influencing bacteriophage attachment to host receptors, or through the damage of the bacteriophage’s structural components (e.g., head, tail envelope), the initiation of lipid loss or nucleic acid damage [17]. For example, Caldeira and Peabody [33] reported on the thermal stability of an RNA phage PP7 (targeting *Pseudomonas*) and found that disulphide bonds between the coat protein dimers stabilised the bacteriophage particle, effectively protecting it against thermal denaturation. However, the ubiquitous distribution of bacteriophages in aquatic environments also allows for the isolation of bacteriophages with specific environmental stability characteristics (Figure 1B). For example, Akhwale et al. [34] reported on the isolation of bacteriophages from a haloalkaline lake with optimum infection capabilities at pH 10–12, while Liu et al. [35] reported on the isolation of bacteriophages from deep-sea hydrothermal fields with stability at 60 °C. Bacteriophages capable of infecting bacteria under varied industrial and environmental conditions (e.g., alkaline pH and high temperature) can then potentially be used for the targeted removal of problem bacteria that flourish in these environments.

## 3. Opportunities for Bacteriophage Biocontrol in the Consumer Water Cycle

As bacteria are present throughout the consumer water cycle (e.g., ARB and DRB in wastewater and biofilm formation in food processing and water distribution systems), bacteriophage biocontrol offers an opportunity to contribute to the bioremediation of natural water sources and the enhancement of various industrial or treatment processes, through the targeted removal of microbial contaminants/pathogens that may be difficult to control using traditional microbial control approaches [11,12,14,26,30].

### 3.1. Natural Water Sources: Agricultural Irrigation and Run-Off Water

At the start of the consumer water cycle, natural water sources may be prone to contamination from various industries due to the inadequate or absent treatment of wastewater [36] (Figure 2). For example, the contamination of surface water sources with nutrient-rich contaminants [e.g., fertiliser runoff (agricultural sector) or raw wastewater] may increase nutrient availability and result in cyanobacterial blooms, which in turn may threaten water security as conventional drinking water treatment processes are inefficient at removing cyanotoxins [37]. Mathieu et al. [38] then indicated that cyanobacterial blooms and toxic cyanobacteria (e.g., *Microcystis aeruginosa*) could be controlled at the source through the use of cyanophages (bacteriophages that specifically infect cyanobacteria) (Figure 2). 

Jiang et al. [39] proceeded to isolate a bacteriophage (Ma-LEP) from a surface water source that specifically targeted *M. aeruginosa*. The interaction of Ma-LEP and *M. aeruginosa* in co-culture was assessed, with results indicating that Ma-LEP significantly impaired the growth and photosynthesis of *M. aeruginosa* and thus had the ability to delay or reduce cyanobacterial bloom intensity. While the bioremediation of environmental water resources may thus be facilitated through the application of bacteriophages for the targeted removal of contaminants of concern, Sharma et al. [40] also highlighted the potential of bacteriophages to indirectly contribute to bioremediation strategies. For example, various bacteria display inherent bioremediation capabilities as they are able to break down environmental contaminants; however, factors including predation and competition from native bacteria may influence the efficiency with which these bacteria can degrade contaminants. Lysogenic bacteriophages may subsequently increase the ability of the bacteria to degrade contaminants, as bacteria infected with these prophages may be more competitive than the native bacteria, due to the competitive advantage the prophage genes infer (including increased immunity to predation by other bacteriophages and bacterial toxins, while remaining mobile enough to reach the toxicants and subsequently degrade them) [40]. As outlined by Sharma et al. [40], stressful environments (e.g., high levels of contaminants) may favour bacterial toxin producers that are able to outcompete toxin-susceptible bacteria. However, the infection of toxin-susceptible bacteria by filamentous bacteriophages increases the ability of these hosts to tolerate toxins, thereby making them more competitive in the environment.

Natural water sources are also closely interlinked with the agricultural sector (including crop, livestock and aqua farming activities), and as such, the use of bacteriophages to target plant (e.g., cherries, onions, melons, potatoes and tomatoes), livestock (e.g., cattle, chicken, pigs and sheep) and aquaculture (e.g., carp, catfish, zebrafish, oysters and shrimp) pathogens [11,13] may not only contribute to preventing economic loss, but also contributes to the improvement of source water quality, as less antibiotics and pesticides will be transferred via the agricultural sector waste streams into the natural water sources. This is of significance, as the leaching of antibiotics and pesticides into natural water sources may lead to the establishment of ARB and DRB in the water where these bacteria may pose a health risk to end-users [41] (Figure 2). The replacement of antibiotics and pesticides in aquaculture was demonstrated by Preenanka et al. [42] where a *Myoviridae* bacteriophage (*Streptococcus* phage-A1) targeting *Streptococcus agalactiae* was used to treat infected Nile tilapia (*Oreochromis niloticus*). In addition, a recent comprehensive review on the use of bacteriophage biocontrol in plants and live animals, by Cristobal-Cueto et al. [11], highlighted that bacteriophage biocontrol in aquaculture currently displays more promise than its application for the treatment of infections in livestock. This may primarily be attributed to the need for more efficient bacteriophage administration techniques in livestock, as certain animal fluids have been shown to be inhibitory to bacteriophages and thus influence treatment efficiency. In comparison, bacteriophages can be added to the source water for biocontrol in aquaculture. Cristobal-Cueto et al. [11] also outlined that the use of bacteriophage treatment cocktails (i.e., use of multiple bacteriophages) could be applied for crop farming, with it being recommended that bacteriophages be administered in the irrigation water to help decrease crop losses caused by pathogenic bacteria.

### 3.2. Water Distribution Systems, the Industrial Sector and Decentralised Water Treatment

Drinking water treatment plants are the primary distributors of water from natural water sources throughout the consumer water cycle and are thus ideal candidates for the implementation of bacteriophage biocontrol strategies (Figure 2). Specifically, bacteriophages may be used to decrease the membrane biofouling of filtration systems, biofouling in distribution systems (i.e., pipes), and for the targeted removal of pathogenic or DRB through their addition to the feedwater/source water entering the system. Moreover, as the direct potable reuse of wastewater has been recommended to improve the consumer water cycle, the addition of bacteriophage biocontrol as an additional treatment intervention may improve the quality of the treated wastewater entering the drinking water treatment plants (Figure 2). For example, Goldman et al. [27] investigated the use of bacteriophages to inhibit biofilm formation on membranes used in ultrafiltration systems for the treatment of sewage effluent. The result showed that the bacteriophage treatment reduced membrane fouling caused by the opportunistic pathogens *Acinetobacter johnsonii*, *Bacillus subtilis* and *P. aeruginosa*, by 40 to 60%. Similarly, Ma et al. [43] demonstrated that the use of bacteriophages facilitated biofouling control during membrane ultrafiltration. The direct spiking of T4 bacteriophages at a concentration of 10^8^ plaque-forming units/mL effectively functioned as a biocidal additive, decreasing the growth of *Escherichia coli* cells in the feedwater while delaying flux reduction in the ultrafiltration membrane for up to 9 h.

Additionally, bacteriophages may be used to control membrane and distribution system biofouling in the industrial sector, as well as for the targeted removal of microbial contaminants and DRB that influence the efficiency of various industrial processes (Figure 2). Various approved [e.g., United States Food and Drug Administration (US FDA), European Food Safety Authority (EFSA), Food Standards Australia New Zealand (FSANZ), amongst others] and commercially available bacteriophage products (e.g., PhageGuard^TM^, ListShield^TM^, AgriPhage^TM^) are currently used as alternative preservatives and antimicrobials in food processing and packaging facilities worldwide [11,44] and the potential use of bacteriophage biocontrol in the food industry will thus not be discussed in the current review. 

Interestingly, bacteriophage biocontrol displays potential to be used within the energy sector for bioremediation and a reduction in occupational health risks. During the extraction and transport of crude oil, water mixes with petroleum and subsequently settles to the bottom of storage tanks. As this drainage water is contaminated with emulsified oil and water-soluble hydrocarbons, it must be treated before being released into the environment. Rosenberg et al. [45] thus investigated the efficiency of a continuous-flow two-stage bioreactor (based on automated chemostat technology) for treating oil refinery drainage water. Analyses of the systems at the outlet points (following treatment) indicated that the highest titre of bacteriophages was observed in the reactor where the highest total organic carbon reduction was recorded, thereby supporting the hypothesis of a bacteriophage-driven microbial loop (rapid production of CO_2_ and the recycling of nitrogen and phosphorous in the environment) that allows for the treatment of oil refinery drainage water.

*Legionella pneumophila* has been identified as an opportunistic pathogen that may be present in various water sources and causes legionellosis, a severe form of pneumonia, upon inhalation of aerosolised water droplets [46]. Subsequently, *L. pneumophila* contamination of fabricated warm water systems in industrial settings, such as cooling towers, has been identified as a potential occupational health risk. However, Lammertyn et al. [46] were the first to report on the isolation and preliminary characterisation of bacteriophages active against *L. pneumophila*, subsequently highlighting the potential of bacteriophage biocontrol of this environmental pathogen. While no additional studies have reported on the isolation of *L. pneumophila* bacteriophages, using genomic analyses of 600 *L. pneumophila* isolates, Deecker et al. [47] recently reported that the *L. pneumophila* isolates analysed in their study were devoid of prophages, and that bacteriophages targeting *L. pneumophila* are most likely lytic gokushoviruses (*Microviridae* family), which are found in various aquatic environments.

While various reviews are available on the use of bacteriophages in the medical sector [21,48,49,50], and these applications are not being focused on in the current review, it is important to note that the use of bacteriophages in the medical industry may reduce the influx of ARB into clinical waste and subsequently wastewater treatment plants, and thereby assist in controlling the spread of antibiotic-resistant genes in the environment (Figure 2). This may primarily be attributed to a phenomenon termed “phage–antibiotic synergy”, whereby antibiotics can increase the susceptibility of bacteria to bacteriophages by weakening the bacterial population, while bacteriophages that interact with bacterial drug efflux systems could in turn restore antibiotic sensitivity in target bacterial populations [25]. 

Recently, Reyneke et al. [30] assessed the use of bacteriophage biocontrol in combination with solar disinfection (a treatment strategy that uses the synergistic effect of solar radiation and solar mild-heat from the sun) to remove *P. aeruginosa* from rainwater, as this opportunistic pathogen is able to initiate various stress response mechanisms and survive conventional water treatment strategies (Figure 2). The results indicate that the bacteriophage pre-treatment sensitised the *P. aeruginosa* to the primary disinfection strategy (i.e., solar disinfection), with gene expression analysis indicating that the *P. aeruginosa* exhibited a decreased ability to initiate stress response mechanisms. Additionally, it was reported that bacteriophage treatment may have reduced *P. aeruginosa* virulence, as a decreased expression of the *P. aeruginosa phzM* virulence gene was also observed for the bacteriophage pre-treated sample. This is significant for the consumer water cycle, as rainwater harvesting has been identified as a potential alternative water source [51]. The use of bacteriophages (e.g., embedded in a filtration device or added directly to a pre-treatment tank) as part of a combination treatment strategy may thus contribute to the provision of a safe alternative water source in urban informal and rural communities [30,52].

### 3.3. Wastewater Treatment

Within wastewater treatment plants, bacteriophages may improve treatment performance by controlling the abundance of key functional microbial groups (e.g., heterotrophic, ammonia-oxidising, nitrite-oxidising, denitrifying and phosphate-accumulating bacteria), through the removal of competing nuisance bacteria such as filamentous bacteria in activated sludge, foaming bacteria that hinder clarification, non-phosphate-accumulating bacteria and sulphate-reducing bacteria, which may decrease the potential methane yield [12]. Additionally, bacteriophages may improve sludge dewaterability and the digestibility of waste activated sludge [12]. Sewage sludge is the biosolid residual material that is produced as a by-product of wastewater treatment processes, and as such its’ effective treatment before release into the environment is crucial. Specifically, microbially produced exopolysaccharides (EPS) facilitate the binding of microbial cells and particulate matter, thereby influencing the formation/settling and water retention ability of sewage sludge. As microbial EPS is primarily composed of water (up to 99%), high levels of this matrix inhibit the dewaterability of both waste activated sludge and anaerobically digested sludge [12]. Bacteriophage polysaccharide depolymerising enzymes have been shown to be effective at degrading EPS in biofilms and may then be used to decrease EPS in sewage sludge and thereby contribute to dewatering [28]. Additionally, the genera *Zoogloea* and *Thauera* have been identified as excessive EPS producers and are the major contributors to dewatering problems in sewage sludge. The introduction of bacteriophages specifically targeting these genera may thus allow for the decreased production of EPS, and thereby improve sludge dewatering [12]. Another challenge that could be addressed using bacteriophages is the biocontrol of filamentous bacteria (e.g., *Microthrix* spp. and *Nocardia* spp.) and foaming bacteria (e.g., *Gordonia* spp. and *Nocardia* spp.), which hinder sewage sludge settling and wastewater clarification [12]. Liu et al. [53] then reported that a combination of four *Siphoviridae* bacteriophages significantly reduced the concentration of *Gordonia* spp. in the wastewater sludge model, as compared to the non-phage reactors. Additionally, continued bacteriophage biocontrol was observed up to nine days after treatment. However, while the use of bacteriophage biocontrol displays promise in various sectors of the consumer water cycle, the dosage/delivery of the bacteriophages in the various environments or systems and the establishment of bacterial bacteriophage resistance are some of the major limitations that need to be overcome to ensure the feasibility of this treatment strategy.

## 4. Limitations and Risks for Bacteriophage Biocontrol

### 4.1. Bacteriophage Resistance

The adaptive nature of bacteria implies that bacteriophage predation may lead to the selection of bacteriophage-resistant bacteria in the environment [54]. However, the bacteria–bacteriophage (host–pathogen) relationship is best described by the antagonistic-coexistence paradigm, with both bacterial bacteriophage resistance mechanisms and bacteriophage counter strategies considered by-products of the relationship [54]. Thus, while bacteria may develop resistance to bacteriophages, the emergence of bacteriophage mutants (bacteriophages able to by-pass bacterial resistance) will ensure the survival of the bacteriophage population [54].

For extensive reviews on the potential resistance mechanisms employed by bacteria against bacteriophages, please refer to Frampton et al. [13], Labrie et al. [16] and Samson et al. [54]. However, as outlined in Figure 3 and Table 1, the primary bacterial bacteriophage resistance mechanisms include (1) the inhibition of bacteriophage adsorption through the modification, masking or variable expression of cell surface receptors, (2) the prevention of nucleic acid entry by host cell proteins into the bacterial membrane, (3) restriction modification systems and (4) the clustered regularly interspaced short palindromic repeats (CRISPR)-CRISPR associated protein systems (CRISPR-Cas) that enable the degradation of foreign bacteriophage DNA, and (5, 6) abortive infection (Abi) systems, which may inhibit various stages of bacteriophage infection or initiate host cell death. 

It is important to note that, amongst other strategies, bacteriophages have been shown to adapt in response to these resistance mechanisms by, for example, modifying their receptor-binding proteins or the restriction sites in their genome (Table 1). Additionally, bacteriophages can avoid the CRISPR-Cas system through mutations or deletions in the protospacer or protospacer-adjacent motif region, or express anti-CRISPR proteins. 

Thus, in order to negate the activation of bacteriophage resistance mechanisms employed by bacteria, bacteriophage treatment strategies could be combined with a secondary treatment (i.e., antibiotics) or bacteriophage cocktails could be utilised [32]. This may increase treatment efficiency and potentially limit the establishment of resistance in the target bacterial population. Additionally, while bacteria may become resistant to bacteriophages, this resistance may be associated with a competitive cost (fitness trade-off), as it has been reported that general bacterial stress response mechanisms or virulence factors may be downregulated in bacteria following exposure to bacteriophages, i.e., bacteriophage-resistant bacteria may become more susceptible to secondary treatment interventions [13,29]. For example, Avrani et al. [55] reported that when *Prochlorococcus* strains developed resistance against T7-like *Podoviridae* bacteriophages through the modification of their cell surface receptors (reduce bacteriophage adsorption), this resistance was associated with a fitness trade-off as the resistant bacteria displayed a reduced growth rate. Additionally, the authors noted that the *Prochlorococcus* strains became susceptible to other bacteriophage strains not used during the initial challenge tests.

### 4.2. Bacterial Target Identification and Bacteriophage Host Range

Apart from bacterial resistance mechanisms, the narrow host range observed for several bacteriophages may hamper the widespread implementation of bacteriophage biocontrol strategies in the consumer water cycle. Therefore, as many bacteriophages have been shown to exhibit a narrow host range (are highly host specific), the target host (i.e., bacterial contaminant or pathogen that needs to be removed) needs to be identified, and in turn should be used for the isolation of the bacteriophage. 

To identify potential target organisms in specific environments, significant progress has been made using next-generation sequencing technologies. However, it may be difficult to identify target organisms in systems or environments that contain complex microbial communities. As outlined by Mathieu et al. [38], it would therefore be necessary to combine metagenomics and other molecular-based technologies that enable the association of phylogeny with function, so as to identify the bacterial target within the system. Additionally, it has been reported that the bacteriophage-to-target-bacteria treatment ratio [multiplicity of infection (MOI) value] needs to be within an optimal range, as a low bacteriophage-to-target-bacteria ratio may allow the bacteria to out-compete the bacteriophages, while a high ratio may accelerate the selection of bacteriophage-resistant bacteria in the population [56]. A narrow bacteriophage host range and insufficient target host density have thus been identified as potential barriers of bacteriophage biocontrol. However, the use of bacteriophage cocktails may potentially increase the target bacteria coverage, while polyvalent bacteriophages can switch between target bacteria based on their density within the specific system. Zhao et al. [57] subsequently compared a polyvalent bacteriophage (*Siphoviridae* bacteriophage ΦYSZ3) and bacteriophage cocktail (*Myoviridae* bacteriophage ΦYSZ1 and *Siphoviridae* bacteriophage ΦYSZ2) treatment to control tetracycline-resistant *E. coli* and chloramphenicol-resistant *P. aeruginosa*. The results show that while both treatment strategies significantly reduced the abundance of the ARB in comparison to the host-specific bacteriophage treatment strategy, the bacteriophage cocktail resulted in the highest treatment efficiency. However, it was also noted that the polyvalent bacteriophage treatment positively influenced the diversity and stability of the bacterial community in the system. 

Lastly, the influence of external environmental conditions (i.e., physiochemical parameters within the environment where the bacteriophage will be applied) on the efficiency of bacteriophage biocontrol strategies (e.g., infection and subsequent replication) is also not well-documented and should be investigated for specific biocontrol applications [13,32].

### 4.3. Bacteriophage Storage and Delivery Mechanisms

The development of efficient methods for bacteriophage production remains a significant hurdle in their large-scale application in biocontrol strategies [58]. While batch reactor systems are considered the most cost-effective production method, they are limited by their maximum production volume and substrate availability. In comparison, continuous reactor systems allow for scalability and control over the bacterial growth rate, which will influence bacteriophage production. Recently, Nabergoj et al. [59] reported on the production of 10^9^ phages/mL/hour in a cellstat system by regulating the dilution rate (via inlet and outlet flux modifications). It has also been recommended that non-pathogenic or attenuated pathogenic hosts be used for bacteriophage production, as this would allow for the safer and more economical production of bacteriophages, due to reduced purification requirements for the removal of pathogenic bacteria [13]. 

Apart from bacteriophage production, the efficient storage and system delivery of bacteriophages is crucial in order to ensure the efficiency of the biocontrol strategy. While most studies have added bacteriophages to feedwater in small-scale trials, this delivery mechanism might not always be suitable for large-scale application in the consumer water cycle. For example, bacteriophage biocontrol may only be required within a specific section of a system (e.g., water distribution system or industrial process), and as such it would not be economically feasible to treat the entire system with the bacteriophage (high treatment concentrations required to ensure delivery of the bacteriophage). Additionally, the physico-chemical parameters within a specific area of the system may be detrimental to the survival of the bacteriophage. Richards and Malik [60] then recently reported on the microencapsulation of bacteriophages in pH-responsive polymer formulations using a membrane emulsification process. The encapsulation of the polymers would offer the bacteriophage protection against exposure to highly acidic conditions (pH 1.5), while the controlled release of the bacteriophages would be triggered at pH 5.5, pH 6 and pH 7, depending on the polymer formulation used. It may thus be practical to encapsulate bacteriophages in a material with a specific release mechanism (e.g., pH, temperature, etc.) to ensure that they are released when required in water distribution systems or industrial processes.

## 5. Genetically Engineered Bacteriophages for Improved Biocontrol Applications 

As outlined in the preceding sections, the use of naturally occurring bacteriophages for biological control applications may be problematic due to several limitations resulting in reduced efficacies [61]. Therefore, the genetic engineering of bacteriophages has been recommended to (1) alter host ranges, (2) improve environmental stability, (3) enhance antibacterial activity, (4) enhance antibiofilm activity and (5) induce lysogenic conversion [62,63,64]. In addition, several legal regulations require attention, including the designation of the therapy to a specific class of medicinal products, and the determination of the appropriate legal framework for the various technical methods of manufacturing and administration of genetically modified bacteriophages [65]. Thus, while genetically engineered bacteriophages exhibiting improved efficacies could potentially be used throughout the consumer water cycle, comprehensive studies are required to assess the safety and efficacy of the genetically engineered bacteriophages prior to their applications as biological control agents. 

### 5.1. Programmable Bacteriophage Host Ranges

Extensive research has been conducted to alter the host range of natural bacteriophages through the engineering of tail fibres and adsorption structures [66]. For example, the homologous recombination of two tail fibre proteins (ORF40 and ORF41) was implemented to modify the host range of a lytic *Acinetobacter baumannii Podoviridae* bacteriophage (ϕAB1) [67]. The ϕAB1tf6 bacteriophage carried the tail fibre protein (ORF40) of ϕAB6 and exhibited lytic activity towards the bacterial host strain (54149) of ϕAB6 but not the original bacterial host strain (M68316). Whilst homologous recombination has allowed for host ranges to be altered to the species or strain level, the approach is labour-intensive and time-consuming, as recombination frequencies are relatively low and often requires the incorporation of selectable markers. 

Genome rebooting has thus been recommended for host range expansion to the genus level [68]. This process refers to the capture of a synthetic bacteriophage genome into *Saccharomyces cerevisiae*, allowing for the genetic manipulation and subsequent reactivation or “rebooting” of bacteriophages in transformed *E. coli* recipient cells [68]. Ando et al. [69] implemented the genome rebooting strategy to engineer a T3 bacteriophage (T3_R(gp17)_) carrying the *Yersinia* phage R tail fibre, which infects both *Yersinia pseudotuberculosis* and *E. coli* strains, using a *Saccharomyces cerevisiae*-based platform. In addition, whole-tail components were exchanged between T7 coliphages and *Klebsiella* K11 bacteriophages, resulting in T7 coliphages with K11 tail components (T7_K11(gp11-12-17)_) and K11 bacteriophages with T7 tail components (K11_T7(gp11-12-17)_). The recombinant K11_T7(gp11-12-17)_ infected *Klebsiella* sp. 380 but not *E. coli* BL21, while the recombinant T7_K11(gp11-12-17)_ infected *E. coli* BL21 but not *Klebsiella* sp. 390. While this approach is currently only limited to Gram-negative bacteria, the yeast-based platform could allow for the modification of the host range to genus level, which could significantly increase the utility of bacteriophage-based biocontrol applications [69].

### 5.2. Bacteriophage Engineering

Physiochemical parameters, including acidity and temperature, have been shown to influence the efficacy of bacteriophages, with several research groups investigating the implementation of genetic engineering or directed evolution for the selection of functional bacteriophages capable of surviving under extreme environmental conditions [70,71,72]. Nobrega et al. [70] synthesised a lipid-coated bacteriophage (T7::PhoE) by fusing the PheE signal peptide to the major capsid protein (10A), which exhibited acid resistance (pH 3.5) and thermostability (42 °C), during an extended incubation period of 1440 min. The approach allows for the generation of bacteriophages that remain active under acidic conditions, which could potentially be used for the eradication of chlorine-resistant bacteria during the treatment of wastewater. More recently, Kering et al. [72] implemented directed evolution by adapting *Myoviridae* bacteriophage Wc4 and *Podoviridae* bacteriophages CX5 and P-PSG-11 through exposure to 60 °C for five consecutive cycles. Subsequently, Favor et al. [71] developed the chemically accelerated viral evolution (CAVE) method consisting of the iterative cycling of chemical mutagenesis and bacteriophage selection for the improvement of physical characteristics. Proof of concept experiments were conducted with T3, T7 and two *Salmonella* bacteriophages (NBSal001 and NBSal002) subjected to 30 and 15 rounds of directed thermal evolution, resulting in thermostability at 60 °C. The CAVE method thus allows for the generation of bacteriophages exhibiting specific physiochemical characteristics, which could significantly increase the application of bacteriophages in the treatment of water under extreme conditions (e.g., high temperature and low pH). 

In addition to improving environmental stability, genetic engineering has been implemented to enhance the antibacterial and antibiofilm activity of bacteriophages [73,74,75]. For example, through the genetic modification of the M13mp18 bacteriophage, Lu and Collins [41], induced the overexpression of the LexA3 repressor of the SOS response in *E. coli* EMG2, increasing fluoroquinolone sensitivity. Concomitant treatment with the Φ_lexA3_ bacteriophage increased the bactericidal activity of ofloxacin by 2.7 and 4.5 orders of magnitude in comparison to the wild-type bacteriophage and no-bacteriophage treatments. Similarly, Edgar et al. [73] genetically modified a lysogenic lambda (λ) bacteriophage with the dominant wild type *rpsL* and *gyrA* genes. Following infection of the *E. coli* host cell with the recombinant λ bacteriophage, dominant wild-type *rpsL* and *gyrA* gene expression resulted in the restoration of antibiotic sensitivity leading to an 8- and 2-fold reduction of streptomycin and nalidixic acid minimum inhibitory concentrations. Although the approach demonstrated by Edgar et al. [73] facilitated the restoration of antibiotic sensitivity (i.e., antibiotic re-sensitisation), it is dependent on the presence of recessive antibiotic resistance genes. Therefore, Yosef et al. [74] developed a lysogenic-lytic strategy to sensitise bacteria to antibiotics and selectively kill antibiotic-resistant bacteria. The authors genetically modified a lysogenic λ bacteriophage to carry and transfer the CRISPR-Cas system (λ_cas_-CRISPR), designed to target plasmid-encoded β-lactamase (*ndm-1* and *ctx-M-15*) genes for degradation, to an antibiotic-resistant *E. coli* resulting in antibiotic re-sensitisation. This approach allows for the selection of antibiotic-sensitive and antibiotic resistance bacteria, which are killed/eliminated by a selected antibiotic and T7 lytic bacteriophage, respectively. Conversely, this method is only effective against plasmid-mediated antibiotic resistance and cannot be implemented for genome-encoded antibiotic resistance [74].

Biofilms, however, remain a major challenge, as the EPS serves as a protective covering or barrier against bacteriophages. Therefore, the development of genetically engineered bacteriophages that produce specific antibiofilm enzymes has attracted growing interest. For example, Lu and Collins [75] genetically engineered a T7 bacteriophage to induce the production of dispersin B (DspB), a polysaccharide depolymerase, following the infection of *E. coli* biofilm cells. The T7_DspB_ bacteriophage reduced bacterial biofilm cell counts by 4.5 (99.997%) orders of magnitude, which is 2 orders of magnitude higher than the reduction recorded for the wild-type T7 bacteriophage. However, the approach is limited as the DspB has a narrow substrate range and is ineffective against multispecies biofilms. Consequently, Pei and Lammas-Samanamud [76] developed a broad-spectrum therapeutic strategy through the exploitation of the bacterial quorum-sensing system. A T7 bacteriophage (T7aiiA) with an incorporated acyl homoserine lactonase gene (*aaiA*) was synthesised and applied to a multispecies biofilm (*P. aeruginosa* PAO1 and *E. coli* TG1 and *E. coli* BL21). This resulted in significant reductions of 65.9% to 74.9%, in comparison to the no-bacteriophage control, and 23.8% to 31.7%, in comparison to the T7 wild-type bacteriophage. 

While most studies focus on lytic bacteriophages, lysogenic bacteriophages are ubiquitous in nature and can be implemented as biological control agents through a process known as lysogenic conversion [61]. Zhang et al. [77] demonstrated lysogenic conversion through the allelic exchange of the *cro* repressor with a nisin-inducible repressor in the *Enterococcus faecalis* bacteriophage ϕEf11. The recombinant ϕEf11(*vir*)^PnisA^ bacteriophage exhibited increased virulence, resulting in the infection of multiple *E. faecalis* strains (33/67; 49%) in comparison to the wild type (4/67; 6%). More recently, Kilcher et al. [78] addressed the limitations of the yeast-based platform (outlined by Ando et al. [69]) by implementing an L-form Gram-positive bacterium (*Listeria monocytogenes*) as the genome recipient and genome rebooting compartment. The yeast-based platform was implemented to induce the conversion of a lysogenic *Listeria* B025 bacteriophage to a lytic bacteriophage, as well as the induction and expression of the peptidoglycan hydrolase enzyme endolysin (ply511), in order to prevent the generation of bacteriophage-resistant mutants. The advanced platform can therefore facilitate the generation of engineered bacteriophages with broad host ranges targeting both Gram-negative and Gram-positive bacteria, which could significantly increase the antimicrobial efficacy of these biological agents.

## 6. Biosafety Considerations for Bacteriophage Biocontrol

While most bacteriophages do not represent a direct threat to human health, the uncontrolled use of large-scale biocontrol in the environment or the use of recombinant bacteriophages may raise certain biosafety concerns, such as the potential dissemination of new genetic traits among bacterial populations or the indirect influence on non-target bacteria, which may influence natural processes in aquatic environments. Adopting a One Health approach, a thorough risk assessment evaluating the properties of bacteriophages used for biocontrol in the consumer water cycle is thus required to protect human health and the environment [79,80]. This approach should collectively aim to protect human health, animal health and environmental health. Additionally, the release of genetically modified organisms (e.g., recombinant bacteriophage) is carefully regulated by governing bodies and a thorough risk assessment of the organism is required before use/release.

The goal of the risk assessment is to define the biological hazard, including the identification of potentially harmful properties of the bacteriophage, through a careful evaluation of the inherent characteristics of the bacteriophage together with any properties acquired due to genetic modification. Additionally, exposure to and the impact on the environment needs to be considered. Specifically, the survival of the bacteriophage, its multiplication and dissemination in the identified ecosystems and the anticipated interaction among the bacteriophage and the organisms (target and non-target) likely to be present in the specific ecosystems need to be determined.

Certain bacteriophages also have the ability to alter the properties of their target host bacteria through “phage lysogenic conversion” (lysogenic bacteriophages integrating genes into the bacterial genome—e.g., toxin genes) or through transduction (transducing bacteriophages that incorporate bacterial genes in the newly formed viral particle and transfer it to a new host where it can recombine with the host bacterial genome) [79]. A crucial step is thus the sequence analysis of the entire bacteriophage genome to reveal gene sequences with significant homology with known phage-encoded toxin genes, or genes encoding bacterial virulence factors that may be transferred by lysogenic bacteriophages [13]. However, while whole genome sequencing costs have decreased in recent years, this analysis may still be cost-prohibitive in developing countries.

From a biosafety point of view, the introduction of a new genetic trait into the bacterial gene pool may have positive, negative, or neutral outcomes, depending on the genes introduced. Most notably, the dissemination of antibiotic resistance through bacteriophage transduction has been demonstrated in multiple antibiotic-resistant bacterial strains, including among bacteria from different species, highlighting the potential risk of the inadvertent conversion of previously non-pathogenic bacteria [79,81]. Moreover, compared to bacteriophages with a narrow host range, broad-host-range bacteriophages may promote genetic diversity and genetic exchange among a wider range of bacterial populations [79]. The determination of the host range of the bacteriophage (especially genetically modified bacteriophages) is thus crucial in the risk assessment process in order to evaluate the probability of the bacteriophage’s propagation in a particular environment, its potential role in global gene transfer and the probability of influencing natural processes in aquatic environments by indirectly influencing key non-target bacteria.

As outlined in the preceding, the environmental stability of bacteriophages is variable, and is dependent on the nature of the specific bacteriophage. Additionally, in aquatic environments, bacteriophages can adsorb onto clay minerals and other particles, which can increase their survival and persistence in terrestrial and aquatic habitats, partly due to protection from UV light [82]. Some bacteriophages can also survive outside their microbial hosts for extended time periods and maintain their ability to infect their bacterial hosts. For example, Waldor et al. [83] reported that shiga-toxin-encoding bacteriophages can survive chlorination and heat treatments, as well as osmotic changes, better than their target bacterial hosts, and subsequently remain infectious in soil for over a month. The survival and persistence of bacteriophages in the environment should therefore be carefully studied to evaluate the extent of potential risks; however, a way in which to minimise potential risks would be to include an additional treatment barrier within the system where the bacteriophage biocontrol is applied. For example, bacteriophages have been shown to be susceptible to natural UV (5% loss of infectivity per hour) [84]. The inclusion of a UV treatment barrier as a post biocontrol intervention may therefore ensure the elimination of any residual bacteriophages remaining in the water after the removal of the target host. 

Overall, based on the biosafety consideration, as compared to lysogenic (temperate) bacteriophages, lytic bacteriophages are: (1) unable to enter into lysogeny and cannot alter the genotype of susceptible bacterial hosts, and (2) are unable to integrate their genetic material inside the bacterial chromosome upon infection, therefore avoiding mutations that could occur during DNA insertion. Additionally, the use of narrow-host-range bacteriophages may limit the risk of their dissemination into the environment.

## 7. Alternative Applications of Bacteriophages to Enhance the Functioning of Water-Based Industries

While the use of bacteriophage biocontrol in the consumer water cycle displays significant potential, various pitfalls need to be addressed before bacteriophages can be applied on a large-scale. Some of the more practical applications of bacteriophages in the consumer water cycle thus include applying them as: (1) water treatment efficiency indicators, (2) water quality indicators or (3) microbial source tracking markers (MST; specifically to identify sources of contamination). 

In comparison to traditional indicator organisms (e.g., *E. coli*, coliforms, etc.), bacteriophages are more resistant to conventional water disinfection strategies (UV radiation, chlorination, etc.), while they are also more accurate surrogates for viral pathogens [85,86,87,88]. Kumlien et al. [86] then highlighted that the global quest for closed urban water cycles will require robust and rigorous treatment monitoring techniques and stressed that implementing bacteriophages to monitor water treatment efficiency will be beneficial in ensuring water safety where wastewater is reused to produce drinking water. Varbanov et al. [89] proceeded to investigate the use of somatic coliphages to monitor wastewater treatment using heat and changes in pH for the removal of severe acute respiratory syndrome coronavirus 2 (SARS-CoV-2). The study found that for somatic coliphages, the T_90_ (time required for a 90% reduction in the viral concentration) at 50 °C was 133 min, while the T_90_ was only 4 min for infectious SARS-CoV-2. It was thus concluded that the thermal and pH treatments that significantly reduced or removed the somatic coliphages from wastewater may be considered effective treatments for the removal of SARS-CoV-2, which was more susceptible to the treatments as compared to the somatic coliphages. 

In contrast to applying bacteriophages as treatment efficiency indicators, bacteriophages that infect intestinal bacteria have been investigated as indicator organisms and MST markers, as they share morphological and biological characteristics with enteric viruses as well as similar fates and survival rates, and they are most likely introduced into the environment via the same route as enteric viruses (i.e., through faecal pollution or wastewater discharge) [87]. The most applied bacteriophages include somatic coliphages (heterogenous bacteriophages capable of infecting coliform bacteria including *E. coli*), F-specific coliphages (capable of infecting coliform bacteria and *E. coli* through sexual pili encoded by the F-plasmid), bacteriophages of *Bacteroides* spp. and enterophages that infect *Enterococcus* spp. More recently, the highly abundant human gut bacteriophage, cross-assembly phage (crAssphage), was also proposed as a faecal indicator and MST marker [90,91].

Ballesté et al. [90] compared bacteriophages previously investigated as MST markers with adenovirus and norovirus to determine which viruses were better suited as indicators of human faecal viruses. The study also assessed whether there were any differences in the sensitivity and specificity of the bacteriophages in different geographical locations, and thus applied them in five European countries, namely, Austria, Germany, Finland, Portugal and Spain. Specifically, bacteriophages infecting human-associated *Bacteroides thetaiotaomicron* strain GA17 (GA17PH), bacteriophages infecting porcine-associated *Bacteroides* strain PG76 (PGPH) and human-associated crAssphage were compared to norovirus GI and GII and human adenovirus. Overall, GA17PH was highly specific and sensitive for human source contamination detection with a specificity of >88% and sensitivity of >83% across all five countries, with no significant geographical differences observed for this marker. In contrast, while crAssphage was present in a higher abundance in the human faecal sources, this marker showed significant geographical variability with 100% specificity for human faecal contamination in Austria, Germany and Finland, but only 38% and 10% specificity in Portugal and Spain. Furthermore, GA17PH was significantly correlated with human adenovirus, crAssphage was significantly correlated with norovirus and adenovirus, while the combination of GA17PH and GA17 correlated with norovirus. The authors ultimately concluded that the bacteriophages were more sensitive for the detection of human contamination, and that the detection of bacteriophages is an easier and more affordable technique for routine analysis [90]. 

Barrios et al. [92] then reported that bacteriophages could also potentially be used as “reporters” for monitoring the circulation of antimicrobial resistance in a specific environment. Specifically, samples were collected from various wastewater treatment plants and screened for β-lactam resistance genes in the bacterial and bacteriophage populations of the samples [92]. Overall, the β-lactam resistance genes were detected in the bacteriophage community as frequently as in the bacterial community; however, the *bla*_CTX-M_ genes were more diverse in the bacteriophage fraction as compared to the bacterial fraction [92]. The authors subsequently recommended that, based on the stability of bacteriophages in wastewater environments and the diversity of resistance genes detected in bacteriophage communities, these viruses could be monitored and analysed to provide information on the antibiotic resistance genes that are or have been circulating in a particular environment [92]. 

## 8. Conclusions

Globally, there is an effort to conserve and protect available water sources, and wastewater reuse and closed consumer water cycles have thus been recommended to ensure the development of sustainable cities. However, as bacteria are ubiquitously distributed throughout the water distribution sector, bacteriophages can potentially be applied throughout the consumer water cycle; from the bioremediation of contaminated natural water sources to drinking water treatment plants, wastewater treatment plants and the treatment of water for irrigation in the agricultural sector, or the treatment of agricultural run-off. However, it is vital that the appropriate bacteriophages be identified for each application with factors such as the target bacteria, the host range of the bacteriophages, and the bacteriophage stability and safety in the environment of interest being primarily considered. 

As bacteria can also develop resistance to bacteriophages, the bacteriophages should ideally be combined with secondary treatment strategies, or bacteriophage cocktails should be applied. The narrow host range of bacteriophages can also be mitigated by isolating polyvalent bacteriophages or by using bacteriophage cocktails. More recently, bacteriophages have been genetically engineered for improved antibacterial applications (e.g., host range modification, improved environmental stability or enhanced activity). However, while this technology displays promise, it is worth noting that (1) bacteriophage capsid capacity limits the size of genetic modifications (i.e., only small DNA fragments can be incorporated), and thus the extent to which a bacteriophage can be modified and (2) the extent to which bacteriophages are genetically engineered may potentially allow for the interaction of these bacteriophages with eukaryotic cells, thus limiting their use in the consumer water cycle due to potential health risks [93]. Thus, while research into the use of bacteriophages is progressing, efficient bacteriophage delivery systems also need to be optimised to ensure the efficiency of this biocontrol strategy, with stimuli-responsive delivery vehicles potentially allowing for a more targeted approach in complex water systems. This approach may then also limit any inadvertent risks to non-target populations, with a thorough risk assessment of the bacteriophage biocontrol application required before use.

Currently, bacteriophages show great potential as faecal contamination indicators, MST markers and treatment efficiency indicators, to monitor the biosafety of water sources and by extension to estimate public health risk. The relatively simple and cost-effective methods required to detect bacteriophages represent a significant benefit in their practical implementation, contributing to the enhancement of the consumer water cycle. However, it has been recommended that as bacteriophages display varying survival rates, the application of a single bacteriophage is not sufficient, and a combination of different bacteriophages should be used as part of a toolbox of MST markers and faecal indicators to accurately monitor water quality and estimate health risk [94]. 

## Figures and Tables

**Table 1:** Mechanism employed by bacteriophages to evade bacterial resistance (adapted from Frampton et al. [13], Labrie et al. [16] and Samson et al. [54]).

Adsorption Inhibition	Injection Blocking	Restriction and Modification	CRISPR/Cas	Abortive Infection
Bacteria may modify their cell surface receptors (mutation), mask receptors or variably express receptors.	Bacterial host cell proteins present on or in the bacterial membrane may prevent the entry of bacteriophage nucleic acids into cell.	Bacterial RM-systems allow restriction endonucleases to cleave foreign invading nucleic acid at a specific recognition site.	CRISPR-Cas systems can recognise and cleave invading foreign bacteriophage nucleic acid.	Bacterial Abi systems may inhibit various steps of the bacteriophage infection process and may also induce host cell death.
In response, bacteriophages may modify their receptor-binding proteins (RBP) to recognise a new bacterial cell surface receptor or produce enzymes to facilitate access to the RBP.	These proteins are normally encoded by superinfection exclusion systems (found in prophages) to prevent infection of bacterial host by multiple bacteriophages.	In response, bacteriophages may modify restriction sites in their genome, decrease restriction sites, degrade co-factors that are required for restriction modification or mask their genome during infection.	In response, bacteriophages can avoid the CRISPR-Cas system through mutations or deletions in the protospacer or protospacer-adjacent motif region or express anti-CRISPR proteins.	Mutations in bacteriophage genes involved in nucleotide metabolism may prevent the activation of the Abi system or allow for the evasion of the Abi system.

## Data Availability

Data sharing is not applicable to this article.
